# Improving Surgical Safety in Living Donor Renal Transplantation With Antiseptic Skin Preparation, Bladder Irrigation, Corner-Saving Vascular Anastomosis, DJ Stenting, and Extravesical Ureteroneocystostomy Modifications: A Comprehensive Approach

**DOI:** 10.7759/cureus.41635

**Published:** 2023-07-10

**Authors:** Shashank Singh, Mohammad S Wani, Arif H Bhat, Abdul R Khawaja, Sajad A Malik, Sajjad A Para, Saqib Mehdi

**Affiliations:** 1 Urology, Sher-i-Kashmir Institute of Medical Sciences (SKIMS), Srinagar, IND

**Keywords:** surgical complications, comprehensive, modifications, abcde, renal transplantation

## Abstract

Introduction

The antiseptic skin preparation, bladder irrigation, corner-saving vascular anastomosis, DJ stenting, and extravesical ureteroneocystostomy (ABCDE) approach encompasses a range of modifications applied during different stages of the surgical procedure in renal transplantation. These modifications include the following: A, antiseptic skin preparation sequentially with cetrimide 3.35%, chlorhexidine scrub 4%, spirit, and povidone-iodine 10%; B, bladder irrigation with amikacin and betadine solution; C, corner-saving end-to-side vascular anastomosis; D, DJ stenting with early postoperative removal within three weeks; and E, extravesical ureteroneocystostomy using our institute's modified Lich-Gregoir technique.

Methods

This prospective observational study was conducted at our institution between March 2021 and May 2023. Data were collected from the patients' medical records and analyzed using Statistical Package for the Social Sciences (SPSS) (IBM SPSS Statistics, Armonk, NY, USA). Statistical tests, including t-test, Mann-Whitney test, chi-square test, and Fisher's exact test, were used for analysis. The study assessed various recipient, donor, intraoperative, and post-transplant factors, as well as surgical complications and stent-related factors.

Results

Out of 72 renal transplantations, 12 (16.6%) had the following surgical complications: urinary (n = 4; 5.5%), wound-related (n = 3; 4.1%), and lymphocele (n = 5; 6.9%). The most common complications were lymphocele (n = 5; 6.9%) and urinary leak (n = 4; 5.5%). Surgical complications were more common in male recipients (91.6% versus 8.3%), as well as in recipients with longer dialysis duration (24 ± 17 versus 11.0 ± 7 months) and had extended hospitalization time (16.4 ± 8.6 versus 8.0 ± 2.9 days) (p < 0.05). Wound infection correlated with longer surgeries (>300 minutes) and other complications. Lymphocele patients had higher drain output (>500 mL) on day 1 and longer hospital stays (>15 days). Urinary tract infections (UTIs) were linked to dialysis duration (>24 months), diabetes, and longer indwelling times of DJ stents and urinary catheters. Early DJ stent removal (<3 weeks) reduced UTI incidence and symptoms (p < 0.05). All complications were categorized as minor (3a or less), according to the Clavien-Dindo classification.

Conclusion

The modified ABCDE surgical approach in renal transplantation decreased the complications, showing favorable outcomes compared to those in the literature.

## Introduction

Renal transplantation is the preferred treatment for end-stage renal disease (ESRD), offering improved quality of life and long-term survival compared to dialysis [1-4]. Living donor renal transplantation presents advantages such as better long-term outcomes and reduced wait times. However, surgical complications can significantly affect patient morbidity and graft survival. Optimizing surgical safety and reducing complication rates are crucial for successful outcomes in living donor renal transplantation.

In this study, we aimed to assess the impact of the modified antiseptic skin preparation, bladder irrigation, corner-saving vascular anastomosis, DJ stenting, and extravesical ureteroneocystostomy (ABCDE) surgical approach on renal transplantation, with the goal of decreasing surgical complications and ultimately improving graft function and patient survival. The ABCDE approach includes modifications at various stages of the procedure, such as antiseptic skin preparation with cetrimide chlorhexidine scrub, spirit, and betadine; bladder irrigation with amikacin and betadine solution; corner-saving end-to-side vascular anastomosis; DJ stenting with early postoperative removal within three weeks; and extravesical ureteroneocystostomy using our institute's modified Lich-Gregoir technique. Previous studies have demonstrated promising results with individual modifications [5-9]. However, the collective impact of the ABCDE approach on overall outcomes remains to be fully elucidated.

Additionally, we evaluated the feasibility and safety of implementing these modifications in routine clinical practice. By investigating the effects of the ABCDE approach, we contributed to the growing body of knowledge on surgical techniques in renal transplantation and provided evidence for optimizing surgical protocols.

## Materials and methods

After obtaining approval from the institutional ethics committee, the Department of Urology at our institute conducted a prospective study from March 2021 to May 2023. The study included patients who underwent kidney transplantation during the specified period and who gave informed consent while excluding those who did not provide consent to participate.

Prior to the surgery, the recipient underwent preoperative evaluations following a standardized proforma. The routine evaluation included consultations with the transplant team, consisting of transplant surgeons, transplant anesthesiologists, transplant nephrologists, immunologists, and clinical nurses from the transplant center. During the preoperative assessment of live donors, medical and surgical suitability for donation was evaluated. Arterial anatomy was assessed using a CT angiogram in all cases. Donor-recipient pairs were subjected to T-cell crossmatching and ensured ABO blood type compatibility. Throughout the assessment process, patients and their families were provided with comprehensive information about transplantation. Preparations for transplant surgery are made after the completion of all medical and legal formalities.

**Figure 1:**
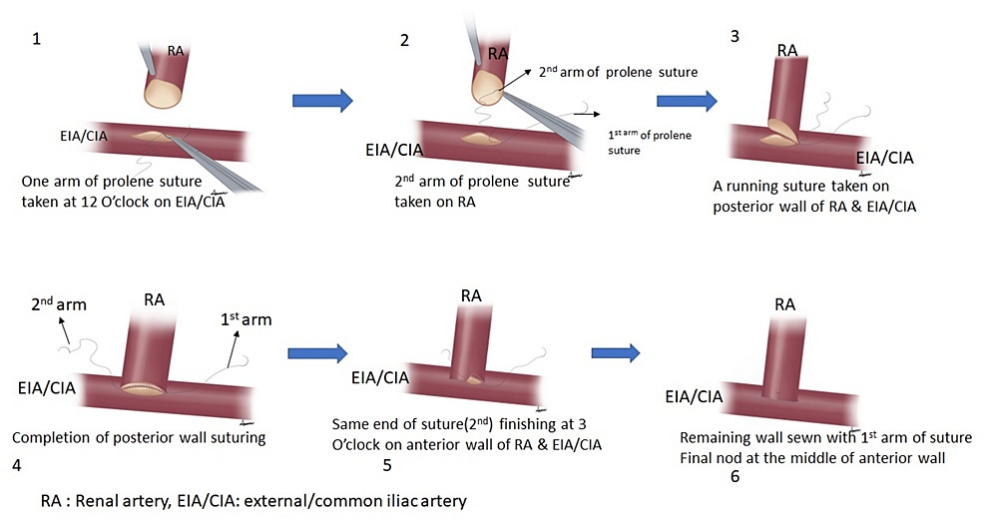
Original conceptual diagram showing "corner-saving end-to-side vascular anastomosis" created specifically for this study RA: renal artery, EIA: external iliac artery, CIA: common iliac artery

**Figure 2:**
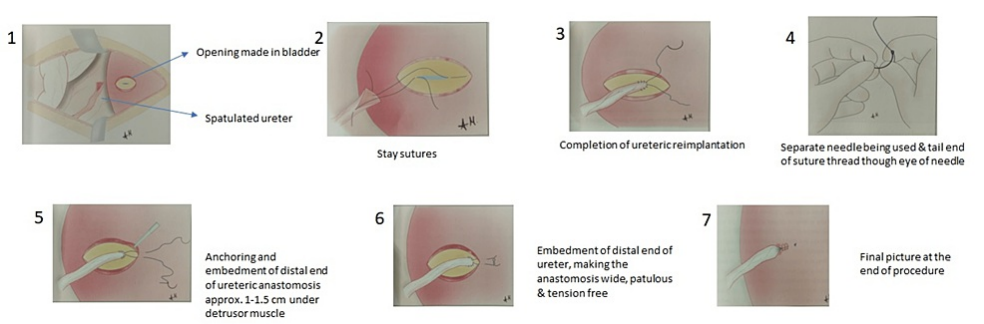
Original conceptual diagram depicting a modified version of Lich-Gregoir ureteroneocystostomy

The harvesting and transplant procedure was done according to standard procedure with various modified surgical approaches in the twin urology operation theaters simultaneously by two surgical teams working together in a coordinated manner. Allograft is harvested and perfused using histidine-tryptophan-ketoglutarate (HTK) solution (custodial). Ex vivo bench surgery was performed wherever required. The modified ABCDE surgical approach was performed during different stages of surgery as follows: A, antiseptic skin preparation involving a sequential application of four solutions, cetrimide 3% (w/v), chlorhexidine scrub 4% (w/v), spirit, and povidone-iodine 10% (w/v), was performed; B, bladder irrigation was performed using a solution of amikacin (1 gm) and betadine (20 mL) in 200 mL of normal saline, introduced into the bladder and retained for 15 minutes by clamping the catheter; C, corner-saving end-to-side vascular anastomosis technique (Figure 1) was employed to ensure secured vessel connection; D, DJ stenting was carried out, and the stent was removed within three weeks post-surgery; and E, extravesical ureteroneocystostomy technique was carried out (Figure 2) using our institute's modified Lich-Gregoir technique.

Post-surgery patients were shifted to the transplant ICU and placed on immunosuppressive therapies based on the transplant recipient's immunological risk and donor factors. All recipients were instructed to follow up for one year in the outpatient clinic on a regular basis. Based on clinical judgment, the stent was removed early (<3 weeks) or late (>3 weeks). A mid-stream urine culture was performed before the stent removal procedure, followed by monthly cultures for up to three months or when the patient exhibited symptoms suggestive of a urinary tract infection (UTI). Univariate analysis of various preoperative, intraoperative, and postoperative variables was done in relation to various surgical complications as well as urinary tract infections in the first three months of the postoperative period using Statistical Package for the Social Sciences (SPSS) version 29 (IBM SPSS Statistics, Armonk, NY, USA), using Fisher's exact test and chi-square test for categorical variables. Student's t-test and Mann-Whitney test were used for continuous parametric and non-parametric variables.

## Results

Out of 72 renal transplantations, the most common underlying diseases causing chronic kidney disease (CKD)/ESRD were CKD (unbiopsied) in 61% of cases and IgA nephropathy in 33% of cases. Other causes, such as focal segmental glomerular sclerosis (FSGS), autosomal dominant polycystic kidney disease (ADPKD), and neurogenic bladder, accounted for 6% of cases. Donor and recipient demographics are shown in Table 1 and Table 2, respectively.

**Table 1:** Summary of donor profile The table presents demographic and clinical information of the kidney donors included in the study. It provides details on gender distribution, age categories, mean age with SD, maximum and minimum donor ages, BMI (>30 kg/m^2^), donor nephrectomy (right or left), and the number of arteries in the donor kidney (single or multiple). SD: standard deviation, BMI: body mass index

Donor	Number (%)
Gender	
Male	29 (40%)
Female	43 (60%)
Age (years)	
<40	26 (36.11%)
40-59	43 (59.72%)
>60	3 (4.1%)
Age (mean ± SD) (years)	48.39 ± 9.5
Maximum donor's age (years)	65
Minimum donor's age (years)	19
BMI (>30 kg/m^2^)	1 (1.3%)
Kidney side	
Right	4
Left	68
Number of arteries in the donor kidney	
Single	66
Multiple	6

**Table 2:** Summary of recipient profile The table provides an overview of the demographic and clinical characteristics of the renal transplant recipients included in the study. It includes information on gender distribution, age categories, mean age with SD, maximum and minimum recipient ages, BMI category (>30 kg/m^2^), the prevalence of comorbidities (hypertension, diabetes, and atherosclerosis), smoking history, previous surgery, time on dialysis (months), second renal transplant, pre-emptive renal transplant, and the timing of stent removal (early (<3 weeks) or late (>3 weeks)). SD: standard deviation, BMI: body mass index

Recipient	Number (%)
Gender	
Female	18 (25%)
Male	54 (75%)
Age (years)	
<40	49 (68%)
40-59	25(32%)
>60	0 (0%)
Age (mean ± SD) (years)	30.92 ± 9.57
Maximum recipient's age (years)	58
Minimum recipient's age (years)	14
BMI (>30 kg/m^2^)	4
Comorbidities (%)	
Hypertension	58 (80.5%)
Diabetes	8 (11.11%)
Atherosclerosis	2 (2.7%)
Smoking history	28 (38.8%)
Previous surgery	3 (4.1%)
Time on dialysis (months)	
<24	52 (72.22 %)
>24	19 (26.38%)
Second renal transplant (left iliac fossa)	1 (1.3%)
Pre-emptive renal transplant	1 (1.3%)
Early stent removal (<3 weeks)	26 (36.11%)
Late stent removal (>3 weeks)	46 (63.8%)

The mean donor warm ischemia time for the transplant procedure was 58.40 ± 1.02 seconds, while the mean cold ischemia time was 32.5 ± 4.45 minutes. The mean anastomosis time (recipient warm ischemia time) was 45.9 ± 4.45 minutes. The modified surgical approach that involved preoperative skin preparation using a four-antiseptic solution, bladder irrigation, and corner-saving end-to-side vascular anastomosis was applied to all recipients. Additionally, extravesical ureteroneocystostomy (our institute's modified Lich-Gregoir technique) with DJ stenting was performed in 70 recipients, and pyeloureterostomy was carried out in two recipients with pelvic ureteric junction obstruction of donor kidney. These modifications were implemented at various stages of the surgical procedure for all recipients. Based on clinical judgment, the stent was removed early (<3 weeks) in 26 recipients and late (>3 weeks) in 46 recipients. Out of 72 recipients, 12 (16.6%) experienced surgical complications. The most common surgical complication was lymphocele, which occurred in 6.9% of patients. Urological complications (5.5%) included ureteral leak in four patients. Wound complications (4.1%) such as wound infection were observed in three patients. None of our patients had reported vascular complications. According to the modified Clavien-Dindo classification for surgical complications, four cases were classified as grade 1 (managed without intervention), three cases as grade 2 (managed with antibiotics), and five cases as grade 3a (managed with radiological intervention in local anesthesia).

Statistical analysis revealed that the average duration of surgery and associated surgical complications were significantly associated with wound complications (p < 0.05) as shown in Table 3. No variable was found to be significantly associated with urological complications (Table 4). The average drain volume on postoperative day 1 and longer hospitalization duration were significantly associated with lymphocele (p < 0.05) as shown in Table 5. Factors such as longer duration of dialysis, diabetes, late removal of DJ stent (>3 weeks), longer dwelling time of the catheter, and DJ stent were significantly associated with urinary tract infections (p < 0.05) as depicted in Table 6.

**Table 3:** Univariate analysis for wound complication The table presents a comparison of various variables between patients who experienced wound complications and those who did not in the study population. The variables include the number of patients in each group, recipient age (mean ± SD), sex distribution, BMI category (>25 kg/m^2^), cause of CKD, comorbidities (specifically T2DM and HTN), previous transplant history, pre-emptive transplant, dialysis dependence, smoking history, associated other surgical complications (lymphocele and urological leak), albumin levels, average duration of surgery (mean ± SD in minutes), and presence of multiple arteries. The table also includes the corresponding P values, indicating the statistical significance of the differences observed between the two groups. SD: standard deviation, BMI: body mass index, CKD: chronic kidney disease, T2DM: type 2 diabetes mellitus, HTN: hypertension

Variables	Wound complication	No wound complications	P value
Number of patients	3	69	
Recipient age (mean ± SD) (years)	48.02 ± 7.2	43.07 ± 8.4	0.3193
Sex (male:female)	2:1	52:17	1.00
BMI (>25 kg/m^2^)	1	5	0.2327
Cause of CKD			
Not biopsied	1	43	0.5559
IgA	2	22	0.2624
Comorbidities (T2DM/HTN)	0/1	8/57	1.00
Previous transplant	0	1	1.00
Pre-emptive transplant	0	1	1.00
Dialysis-dependent transplant	3	68	
Smoking history	1	27	1.00
Associated other surgical complications (lymphocele + urological leak = 9)	2	7	0.0394
Albumin (g/dL)	3.1 ± 0.3	3.4 ± 0.7	0.4646
Average duration of surgery (mean ± SD) (minutes)	302.00 ± 15.87	280 ± 10.4	0.0008
Multiple arteries	1	5	0.2327

**Table 4:** Univariate analysis for urological complications The table presents a comparison of various variables between recipients who experienced urinary leak and those who did not in the study population. The variables include recipient age (mean ± SD), sex distribution, donor age (mean ± SD), presence of multiple arteries, presence of a single renal artery, previous transplant history, cold ischemia time (mean ± SD in minutes), use of our institute's modified Lich-Gregoir ureteroneocystostomy technique, presence of pyeloureterostomy, early stent removal (DJ stent) within three weeks, and late stent removal (DJ stent) after three weeks. The table also includes the corresponding P values, indicating the statistical significance of the differences observed between the two groups. SD: standard deviation

Variables	Urinary leak (n = 4)	No urine leak (n = 68)	P value
Recipient age (mean ± SD) (years)	34.5 ±11.5	30.5 ± 12.5	0.5346
Sex (male:female)	3:1	51:17	0.7873
Donor age (mean ± SD) (years)	37.5 ± 7.5	32.5 ± 5.2	0.4332
Multiple arteries	0	6	1.00
Single renal artery	4	64	
Previous transplant	0	1	1.00
Cold ischemia time (mean ± SD) (minutes)	21.44 ± 4.3	21.66 ± 3.9	0.8977
Our institute's modified Lich-Gregoir ureteroneocystostomy	4	66	1.00
Pyeloureterostomy	0	2	
Early stent removal (DJ stent) (<3 weeks)	2	24	0.6164
Late stent removal (DJ stent) (>3 weeks)	2	44	

**Table 5:** Univariate analysis for lymphocele The table presents a comparison of various variables between recipients who developed lymphocele and those who did not in the study population. The variables include recipient age (mean ± SD), sex distribution, BMI (>25 kg/m^2^), presence of DM, presence of HTN, type of donor (live related or live unrelated), presence of multiple graft renal arteries anastomosis, cold ischemia time (mean ± SD in minutes), anastomosis time (mean ± SD in minutes), total surgery time (mean ± SD in minutes), postoperative creatinine at one month (mean ± SD), drain volume on POD 1 (mean ± SD in mL), drain volume on POD 5 (mean ± SD in mL), drain removal duration (mean ± SD in days), and hospitalization duration (mean ± SD in days). The table also includes the corresponding P values, indicating the statistical significance of the differences observed between the two groups. SD: standard deviation, BMI: body mass index, DM: diabetes mellitus, HTN: hypertension, POD: postoperative day

Variables	Lymphocele (n = 5)	No lymphocele (n = 67)	P value
Recipient age (mean ± SD) (years)	44.2 ± 6.25	42.5 ± 7.2	0.6096
Sex (male:female)	4:0	50:18	0.5660
BMI (>25 kg/m^2^)	1	5	0.3613
DM	0	8	1.00
HTN	3	56	0.2480
Live related donor transplant (n = 56)	3	53	0.3070
Live unrelated donor transplant (n = 16)	1	15	
Multiple graft renal arteries anastomosis	0	6	1.00
Cold ischemia time (minutes) (mean ± SD) (minutes)	29.5 + 2.45	32.0 + 4.05	0.1081
Anastomosis time (mean ± SD) (minutes)	48.19 ± 5.1	45.91 ± 4.2	0.2519
Total surgery time (mean ± SD) (minutes)	329.6 ± 23	302.3 ± 31	0.0585
Postoperative creatinine at one month (mean ± SD)	1.22 ± 1.11	1.48 ± 1.7	0.7383
POD 1 drain volume (mean ± SD) (mL)	549.00 ± 293.99	240 .43 ± 236.64	0.0072
POD 5 drain volume (mean ± SD) (mL)	138 ± 85.96	89.42 ± 50.95	0.0544
Drain removed (mean ± SD) (days)	8.00 ± 4.89	6.70 ± 1.34	0.1134
Hospitalization duration (mean ± SD) (days)	17.2 ± 4.5	8.9 ± 2.9	<0.01

**Table 6:** Univariate analysis for UTI The table provides a comparison of various variables between recipients who experienced UTI during the three-month follow-up period and those who did not in the study population. The variables include recipient age (mean ± SD), sex distribution, dialysis duration (mean ± SD in months), donor age (mean ± SD), donor sex distribution, presence of diabetes, history of previous recurrent UTI, occurrence of urological complications, timing of stent removal (early or late), duration of DJ stent placement (mean ± SD in days), urinary catheter dwelling time (mean ± SD in days), presence of urinary tract abnormalities (such as reflux bladder dysfunction or neurogenic bladder), and history of prior urological operation. The table also includes the corresponding P values, indicating the statistical significance of the observed differences between the two groups. UTI: urinary tract infection, SD: standard deviation, CKD: chronic kidney disease

Variables	UTI present (n = 8) in the follow-up period of three months	UTI absent (n = 64) in the follow-up period of three months	P value
Recipient age (mean ± SD) (years)	47.5 ± 7.2	41.7 ± 9.5	0.1006
Sex (male:female)	4:4	50:14	0.1010
Dialysis duration (mean ± SD) (months)	28 ± 09	12 ± 5	<0.0.01
Donor age (mean ± SD) (years)	47.8 ± 10.9	45 ± 11.3	0.5095
Donor sex (male:female)	7:1	47:17	0.6699
Diabetes	3	5	0.0396
Previous recurrent UTI	1	4	0.4551
Urological complications	2 (25%)	2 (3.1%)	0.0584
Early stent removal (<3 weeks) (n = 26)	0	26	0.0444
Late stent removal (>3 weeks) (n = 46)	8	38	
DJ stent (mean ± SD)	28.63 ± 3.85	21.34 ± 2.5	<0.01
Urinary catheter dwelling time (mean ± SD) (days)	8.9 ± 4.3	6.9 ± 1.4	0.0065
Urinary tract abnormalities (reflux bladder dysfunction and neurogenic bladder)	0	2	1.00
Prior urological operation	0	1	1.00

## Discussion

A comparison was made between the percentages reported in the literature and the corresponding findings from our study. In Table 7, a comprehensive review of relevant literature is presented, highlighting the reported percentages of surgical complications, wound complications, urological complications, lymphocele, and early urinary tract infections (UTI) in previous studies.

**Table 7:** Review of literature and comparison with our study The table provides a comparison of adverse events, including overall surgical complications, wound complications, urological complications, lymphocele, and early UTI within a three-month period between previous studies and our study. The adverse events reported in previous studies are cited with the corresponding authors, publication year, and the reported percentage. Our study's findings for each adverse event are also presented for comparison. UTI: urinary tract infection

Adverse events	Previous studies	Year (published)	Percentage	Our study
Surgical complications	Carvalho et al. [10]	2019	16.9%	16.6%
	Lempinen et al. [11]	2015	15.5%	
	Pillot et al. [12]	2012	24.5%	
Wound complications	Peluso et al. [13]	2020	15.7%	4.1%
	Lau et al. [14]	2019	7.7%	
	Santangelo et al. [15]	2009	15.43%	
	Humar et al. [16]	2001	4.8%	
Urological complications	Bruintjes et al. [17]	2019	6.2%	5.5%
	Buresley et al. [18]	2008	4.8%	
	Neri et al. [19]	2009	8.7%	
	Dinckan et al. [20]	2007	6.01%	
Lymphocele	Atray et al. [21]	2004	24%	6.9%
	Haberal et al. [22]	2016	4.2%	
Early UTI (three months)	Senger et al. [23]	2007	<30%	11.11%
	Abbott et al. [24]	2004	17%	

Twelve (16.6%) recipients in our study developed surgical complications, which is in accordance with international data published [10-12], where the authors reported surgical complications in approximately 5%-20% of cases.

Renal transplantation is considered a clean-contaminated case; therefore, preventing surgical site infection (SSI) by properly preparing the skin before incision is a vital part of the overall care given to recipients. We used four antiseptic solutions for preoperative skin preparation, i.e., cetrimide 3% (w/v), chlorhexidine scrub 4% (w/v), spirit, and povidone-iodine 10% (w/v) serially. This step helped us to reduce some amount of SSI/wound complications in renal transplantation. In our study, the incidence of wound complication in recipients was 4.1%, which was less than 7.7%-15.7% as mentioned in the literature [13-16].

Urological complications following kidney transplantation varied between 4.2% and 14.1% in early studies (1970-1990s) and decreased from 3.7% to 6% in later studies (1990-2000). The incidence of urine leaks ranged from 1.5% to 6% in studies conducted during the 1990s to 2000s [25]. These variations can be attributed to different transplantation eras, diagnostic tools, and surgical expertise. Importantly, the incidence of urological complications significantly reduced with increased center experience. In our study, we have only four (5.5%) cases of urine leak as urological complications, which was comparable to previous studies [17-20].

Lymphoceles have been reported to occur in 4.2%-24% of transplants as per literature [21,22], but we encounter only 6.9%.

As the most common site of UTI after transplant is the urinary bladder, dysfunctional and unused urinary bladder in oliguric/anuric patients with ureteric stent in situ increases the chances of UTI after transplantation. It happens due to increased detrusor pressure in an unstable postoperative bladder with stent abolishing ureteric peristalsis and dilating the vesicoureteric junction. Considering this rationale, we used bladder irrigation with amikacin 1 gm with betadine 20 mL in 200 mL normal saline. This approach provides the advantage of targeted treatment with high efficacy, reduced systemic side effects, and preserved renal function. In our study, eight (11.11%) cases developed UTI, which was much less than in the literature, i.e., 17% [23,24], in the initial three months with the most common pathogen found to be *E. coli*.

Earlier stent removal (<3 weeks) was associated with decreased incidence of UTI (Table 6) and did not show a higher incidence of urinary leakage (Table 4) compared to late removal (>3 weeks), which was similar to previous systematic review [26] and Cochrane analysis [9].

As we all know, the proven advantage of extravesical ureteroneocystostomy Lich-Gregoir is undeniable [27,28]. We have modified the Lich-Gregoir technique (Figure 2). This involves anchoring and embedment of the distal end of ureteric anastomosis approximately 1-1.5 cm under the detrusor muscle. We use a separate needle for threading the sutures under the seromuscular layer. This step reduces the tension on the anastomotic site and makes it more patulous and thus prevents puckering at the ureteroneocystostomy site. Also, DJ stenting is done in all cases of renal transplant surgeries at our center. This helps in giving rest to the anastomotic site and improves the outcome by reducing major urological complications [8].

In our study, we did not encounter any vascular complication in all 72 renal transplant recipients who underwent our corner-saving end-to-side renal vascular anastomosis technique as described in Figure 1. This may occur due to a short sample size. However, we believe that this technique is a safe and simple approach to performing renal vascular anastomosis that will decrease the rate of vascular complications. By avoiding the final knot at the corner, which is a potential weak point, as the pressure of blood passing through the corner may place additional stress on the suture line of vascular anastomosis, we are able to decrease the chances of leaking.

Despite the valuable insights gained from this study, several limitations should be acknowledged. Firstly, the small sample size of 72 renal transplant recipients restricts the generalizability of the findings. A larger sample size would provide more robust data and enhance the applicability of the results to a broader population. Secondly, it is important to consider that this study was conducted at a single center, which may introduce bias and limit the generalizability of the findings to other settings. The use of different surgical techniques, variations in patient characteristics, and diverse protocols across centers could influence the outcomes observed.

Another limitation is the lack of long-term follow-up data, as this study focused primarily on short-term outcomes. Understanding the impact of surgical complications on graft survival and patient outcomes over an extended period is crucial for comprehensive evaluation. Future studies should incorporate long-term follow-up to address this limitation. Additionally, it should be noted that this study specifically focused on living donor renal transplantation and did not include deceased donor transplantation. Furthermore, the study did not explore the outcomes associated with minimally invasive approaches, such as robotic or laparoscopic-assisted renal transplantation. Considering these factors in future research would provide a more comprehensive understanding of the benefits and limitations of different surgical techniques in renal transplantation. Lastly, certain variables that may influence surgical complications, such as individual surgeon experience and variations in specific surgical techniques, were not accounted for in the analysis. While uniform surgical techniques were employed for all recipients in this study, future investigations could incorporate these variables to gain a more in-depth understanding of their impact on surgical outcomes in renal transplantation.

## Conclusions

The modified antiseptic skin preparation sequentially with cetrimide 3.35%, chlorhexidine scrub 4%, spirit, and povidone-iodine 10%, bladder irrigation with amikacin and betadine solution, corner-saving end-to-side vascular anastomosis, DJ stenting with early postoperative removal within three weeks, and extravesical ureteroneocystostomy using our institute's modified Lich-Gregoir technique (ABCDE) surgical approach in renal transplantation contributed to a reduction in surgical complications at our hospital. The observed complication rate in our study was comparable to or lower than that reported in various literature studies. These findings suggest a positive impact of these modifications on enhancing the surgical safety of renal transplant recipients. However, further validation through larger and comparative studies would be necessary to strengthen these observations.
